# Presence and persistence of hepatitis E virus RNA and proteins in human bone marrow

**DOI:** 10.1080/22221751.2020.1761762

**Published:** 2020-05-18

**Authors:** Lin Wang, Li Yan, Jieling Jiang, Yuyi Zhang, Qiyu He, Hui Zhuang, Ling Wang

**Affiliations:** a Department of Microbiology and Infectious Disease Centre, School of Basic Medical Sciences, Peking University Health Science Centre, Beijing, People’s Republic of China; b Department of Severe Hepatology, Shanghai Public Health Clinical Centre, Fudan University, Shanghai, People’s Republic of China; c Department of Hematology, Shanghai General Hospital, Shanghai Jiao Tong University School of Medicine, Shanghai, People’s Republic of China

**Keywords:** Hepatitis E virus, hepatitis E, extra-hepatic replication, bone marrow

## Abstract

Hepatitis E virus (HEV) infection is primarily manifesting as acute hepatitis, but extra-hepatic replication and injury are frequently reported. During the study period, we discovered two acute myeloid leukaemia (AML) patients infected with HEV genotype 3 and 4, respectively, and HEV RNA and/or viral proteins were persistently detected in the bone marrow of both patients. The finding suggests that HEV can replicate in human bone marrow as it may serve as a new target site and reservoir of HEV persistence.

Hepatitis E virus (HEV) is a major cause of acute viral hepatitis worldwide and it is estimated that one-third of the world population have been exposed to HEV [1]. The virus typically induces self-limiting disease in immunocompetent individuals. However, HEV-infected immunocompromised patients, such as solid organ transplant recipients, HIV/AIDS patients and cancer patients receiving chemotherapy are prone to develop chronic hepatitis E [1]. In addition, increasing studies reported that a wide range of extra-hepatic manifestations are associated with HEV infection and HEV RNA can be detected in many organs and body fluids, including urine, breast milk and cerebrospinal fluid [2].

Bone marrow is one of the most important organs of human. Many pathogens, including *Leishmania* [3], Epstein–Barr virus (EBV) [4], parvovirus [5] and human pegivirus [6], can be detected in bone marrow and sometimes causing relevant haematologic diseases. To date, direct detection of HEV in human bone marrow has never been documented. Here, we present 2 cases of HEV-infected acute myeloid leukaemia (AML) patients with persistent detection of HEV RNA and/or viral proteins in bone marrow.

All the samples collected from the patients were screened for the presence of HEV RNA. The present study was performed in accordance with the Helsinki Declaration and all patients gave informed consent and permission for testing of clinical samples. RNA was extracted from 100 μL of clarified faecal suspension supernatant, serum and bone marrow fluid using TRIzol reagent (Invitrogen, Burlington). A heminested reverse transcription-PCR, targeting ORF1 of the HEV genome, was used to screen for the presence of HEV RNA [7]. HEV-positive samples were commercially sequenced according to the manufacturer’s instructions (Beijing Genomics Institute, Beijing). All sequences were submitted to GenBank with accession numbers MF996356 and MT110146. Quantification of HEV RNA was carried out using a commercial One-Step RT-qPCR kit (A6120; Promega, USA) and method that has been previously described [7].

For Immunohistochemistry (IHC), bone marrow biopsy and smears were prepared for IHC (microscope equipped with a digital camera, Olympus CX31, Japan) and by first being fixed in 10% neutral buffered formalin immediately following sampling according to the literature [7]. HEV proteins were visualized by using HEV ORF2-specific polyclonal antibodies (bs-15457r, Bioss, Woburn).

During May 2017 to Mar 2018, we received request for HEV RNA detection of serum and faecal samples from 2 AML patients. Patient 1 is a 22-year-old male with AML diagnosed in Jan 2017. In May 2017, he was found with an abnormal liver function, alanine aminotransferase (ALT) 228.9 U/L during his routine examination. Patient 2 is a 29-year-old female diagnosed with AML in Jul 2017 and was planned to perform bone marrow transplantation in Mar 2018. However, in Feb 2018 she was found with an abnormal ALT level, 150.0 U/L in her preoperative evaluation. Both patients were treated with chemotherapy. Jaundice was not observed at admission. Current infection of hepatitis A, B, C virus, human immunodeficiency virus, EBV and cytomegalovirus were ruled out. Liver-directed autoantibodies were all negative. However, anti-HEV IgM and IgG were tested positive (Wantai, Beijing). HEV RNA was subsequently detected in the serum and faecal samples in our laboratory. Sequencing of partial HEV ORF1 genome and subsequent analysis has revealed that Patient 1 and 2 were infected with HEV genotype 3 and 4, respectively. Serum and faecal samples of Patient 1 were collected monthly from May 2017 to Oct 2017. HEV RNA was tested positive in serum and faecal samples from May 2017 to Sep 2017. Beginning on Aug 23rd, oral ribavirin was administrated at the dose of 1000 mg per day and HEV RNA became undetectable on Oct 2017. Serum and faecal samples of Patient 2 were only available at admission on Mar 2018. Overview of patients’ samples collection, screening and results, and timelines of major clinical events were summarized in Supplementary Figure 1.

By literature searching, we found no direct evidence of HEV presence or replication in the human bone marrow. Bone marrow fluid samples were regularly collected by acupuncture due to routine assessment of disease progress of AML and the remained samples from Patient 1 were available on Aug 21st and Sep 14^th^ 2017. HEV RNA was detected in the bone marrow fluid at a titer of 1.44×10^5^ copeis/mL and 3.03×10^3^ copeis/mL, respectively. The viral RNA titer of serum sample collected on Aug 21st was 3.75×10^4^ copeis/mL, relatively lower than that of the bone marrow fluid. Bone marrow biopsy was performed on Sep 14th for Patient 1 and we found positive stains of HEV ORF2 proteins in bone marrow cells by IHC (Figure 1A). To further investigate the presence of HEV in human bone marrow, we retrospectively obtained available bone marrow smears from both patients and a smear from a HEV-negative patient as negative control. For Patient 1, HEV ORF2 positive stains were detected from May 2017, the time of the onset of HEV infection, to Jul 2017. Smears made on Feb 2017, 3 months before HEV infection onset, and Feb 2018, 4 months after HEV infection cleared, were negative (Figure 1A). For Patient 2, bone marrow smears were available on Dec 2017 and Feb 2018 and were both positive for HEV ORF2 proteins (Figure 1B). The positive stains were mainly observed in granulocytes and sometimes spread through the cytoplasm or concentrate in the nucleus. No obvious positive labelling was observed in red blood cells (Figure 1).

**Figure 1.**
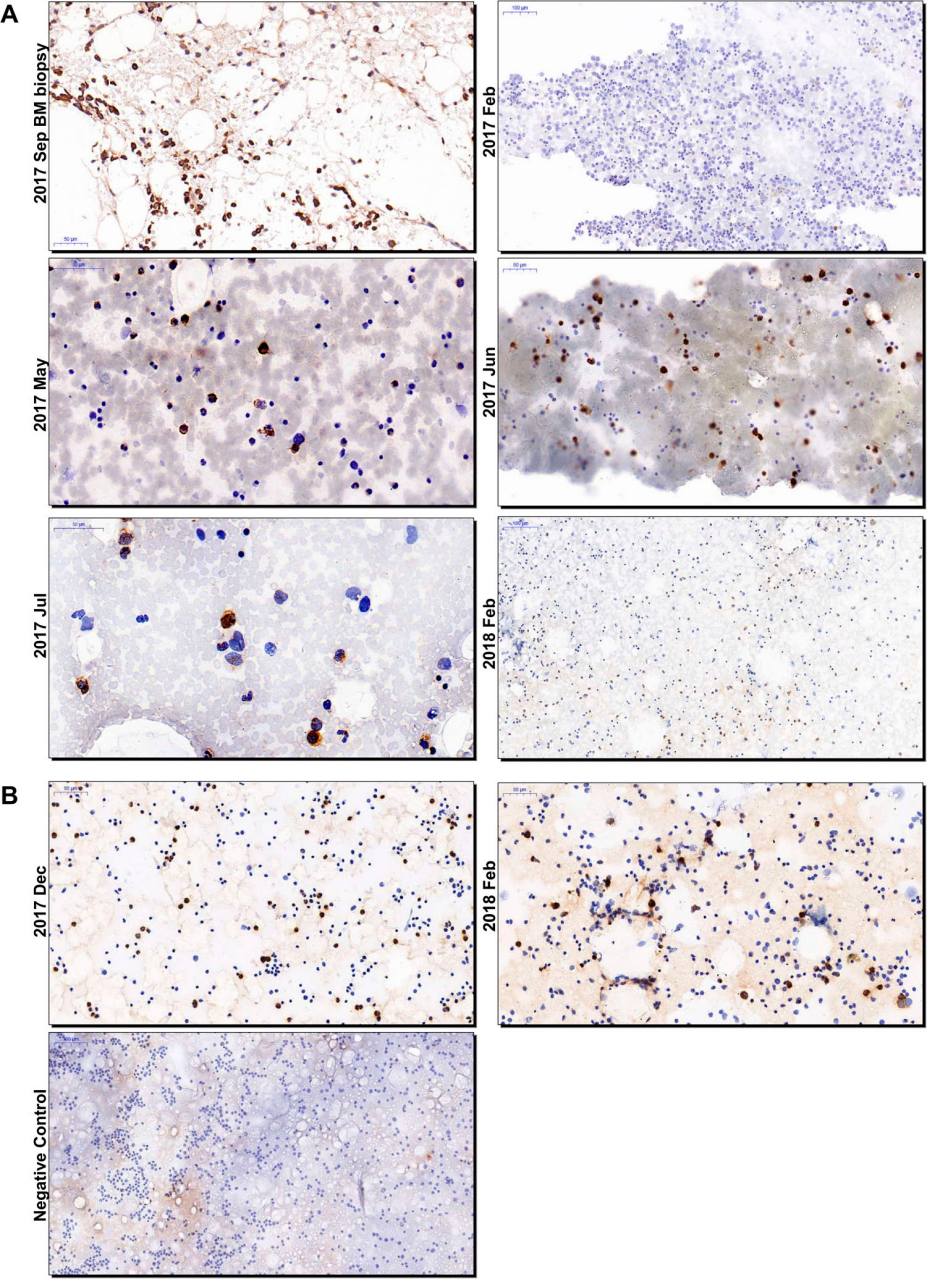
Immunohistochemistry analysis of HEV ORF2 proteins in bone marrow smears and biopsy of patients. Photos of bone marrow smears and bone marrow biopsy collected from Patient 1 (A) and 2 (B) were showed. The time of collection was designated on the left side of each photo. Bars, 50 or 100 μm. HEV-specific antibodies (bs-15457r; Bioss, Woburn) were used. The secondary antibody used for staining was goat anti-rabbit IgG (Goodbio Technology, Wuhan). ORF2, open reading frame 2; HEV, hepatitis E virus; BM, bone marrow.

Among many extra-hepatic tissues and organs, HEV has never been detected in human bone marrow. In this study, we demonstrated that HEV RNA and proteins can be found in bone marrow. The presence and persistence of HEV RNA and viral proteins in bone marrow samples correlates well in timing with the disease course of HEV infection. Moreover, the findings suggest that the bone marrow tropism of HEV can be found with both HEV genotype 3 and 4.

The constant detection of HEV ORF2 proteins, the capsid proteins, in bone marrow suggests that HEV may replicate in the bone marrow cells. Very recently, an animal study found that HEV antigens can be detected in the bone marrow of both HEV genotype 3 experimentally infected or naturally infected cynomolgus monkeys [8]. The present study, along with the previous study conducted in cynomolgus monkeys, reinforces the assumption of bone marrow as a new HEV target. However, certain limitation still exists in our study and the direct HEV infection using cultured bone marrow cells is warranted in the future in order to help us understand more about the replication of HEV in the bone marrow.

HEV can replicate in many extra-hepatic human organs or tissues in vivo or ex vivo, including placenta [9], endothelial cells [10] and intestines [11]. Sometimes the virus can induce extra-hepatic manifestations. HEV-associated haematologic diseases, such as thrombocytopenia, monoclonal gammopathy and haemolytic anaemia, have been reported [2]. The role of HEV infection in such diseases should be investigated. Bone marrow samples should be tested for HEV markers and histology in order to clarify whether HEV will directly damage the bone marrow and subsequently induce disease. Furthermore, as transfusion-transmitted HEV infection has increased in recent years, the possibility of transmission of hepatitis E through bone marrow transplantation cannot be ignored and warrants clinical attention [12]. The bone marrow may serve as a hidden viral target and reservoir of HEV. Therefore, HEV-infected patients with haematologic diseases or malignancies should be routinely tested for HEV markers in bone marrow samples.

## Supplementary Material

Supplemental Material
