# Supplementary figures and images for: Presence and persistence of hepatitis E virus RNA and proteins in human bone marrow
Source: Emerg Microbes Infect. 2020 May 18;9(1):994–7. doi: 10.1080/22221751.2020.1761762 (PMC7269076; doi:10.1080/22221751.2020.1761762)

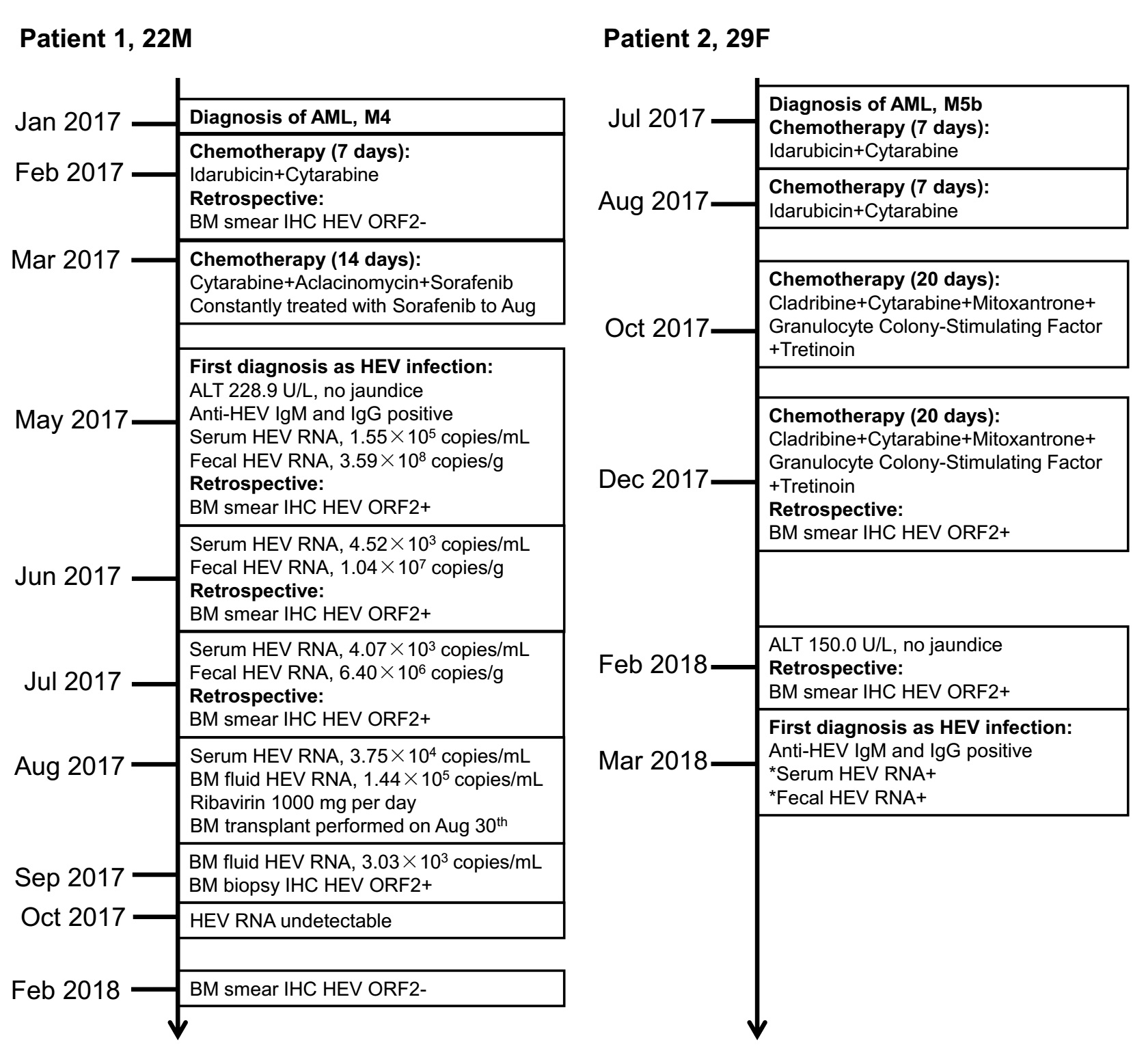

Supplement: Supplemental Material [file TEMI_A_1761762_SM5963.jpg]
